# Report of Diseases in the Public and Private Practice of One of the Physicians of the Finsbury Dispensary, from the 20th of June, to the 20th of July

**Published:** 1805-08-01

**Authors:** J. Reid

**Affiliations:** Greville Street, Brunswick Square


*Report of Diseases in the public and private Practice of one of the Physicians of the Finsbury Dispensary, from the 20th of June, to the 20th of July.*

| | | | |
|---|---|---|---|
| Febris - - - - - | 3 | Menorrhagia - - - | 1 |
| Catarrhus - - - - | 5 | Leucorrhœa - - - | 3 |
| Dyspnœa ebriosa - - | 1 | Anasarca - - - - | 6 |
| Phthisis pulmonalis - | 7 | Epilepsia - - - - | 2 |
| Cynanche - - - - | 9 | Morbi Infantiles - - | 16 |
| Ophthalmia - - - - | 4 | Diarrhœa et Cholera - | 12 |
| Dyspepsia - - - - | 18 | Morbi Cutanei - - - | 9 |
| Hypochondriasis - - | 2 | | |

The Reporter has this last month been impressed more deeply than ever he was before with the fatal folly of bleeding in the generality of those cases to which venesection is too frequently applied.

A person who, at a very advanced period of life, was sinking under the combined operation of age and intemperance, was advised, on account of a difficulty of breathing, arising from general debility and a mutilation of the pulmonary organs, to experience frequent and extravagant evacuations from the arm, which, of course, in a very short time, put a period to his terrestrial existence.

If the employment of the lancet, although in some instances it is of undoubted use and necessity, were abolished altogether, it would, perhaps, save annually a greater number of lives than in any year the sword has ever destroyed.

Medical men are sometimes apt to consider themselves, and are generally regarded by others, as insignificant and inefficient, unless they are doing something; that is, either performing some painful operation, or administering some powerful remedy. Whereas, the fact is, that, in no inconsiderable proportion of cases, the best thing that can be done is to let the patient alone. An inflammatory fever,* or a habit indicating excess of general excitement in this enervated age, very rarely indeed occurs. And *local* inflammations, such as acute rheumatism, gout, or cynanche, will seldom, with impunity, permit the opening of a vein. In the last disease, the writer has had more especial reason to entertain this opinion, in which he is confirmed by the authority of a man celebrated as a philosopher, altho' not a member of the medical profession.

"Ah! these accursed physicians, they will certainly kill her with their blood-lettings. I have been myself extremely subject to the quinzy, and have invariably found that bleeding increased its violence: when, on the other hand, I contented myself with simply using a gargle, and putting my feet in warm water, I generally found myself well the following day."†

In cases of scrophulous ophthalmia, the writer has recently found advantage in applying *cold* to the whole body, as well as to the organ more particularly affected; the salutary power of this agent seems to increase nearly in proportion to the extent of surface to which it is applied.‡

Fevers, and other analogous complaints, appear not to prevail at present, to that extent which might be expected in London, at this period of the summer. It is not the intensity of the heat, so much as the complicated polution with which, in consequence of it, the atmosphere of the metropolis is, more especially in the warmer months, impregnated, that tends to disorder and to debilitate the constitution of its inhabitants.

"It is not air, but floats a nauseous mass
Of all obscene, corrupt, offensive things." §

Happy are they, who, unconfined by professional or any other chains, are, at this season of the year, at liberty to enjoy the salutary fragrance of vegetation, or to seek refreshment and relief in the still more enlivening breezes and invigorating exhalations of the sea.

*Greville Street, Brunswick Square, July 25, 1805.* J. REID.

---

* Dr. Cullen states, that he never saw an instance of this fever during forty years of the most widely extended practice.

† Original Correspondence of Rousseau.

‡ The Reporter has lately received a copy of a Treatise on the Operation of Cold from Dr. Stork of Bristol, a writer of merit, who, although not an implicit disciple, exhibits, in his work, a mind illumined with the rays of Brunonian philosophy.

§ Armstrong.

